# Malignant Transformation of Endometrioma in a Woman with a History of Ovulation Induction and In Vitro Fertilization

**DOI:** 10.1155/2012/497362

**Published:** 2012-12-04

**Authors:** Terri L. Woodard, Awoniyi O. Awonuga, Elizabeth Puscheck

**Affiliations:** ^1^School of Medicine, Wayne State University, 60 West Hancock Street, Detroit, MI 48201, USA; ^2^Division of Reproductive Endocrinology and Infertility, Department of Obstetrics and Gynecology, School of Medicine, Wayne State University, 60 West Hancock Street, Detroit, MI 48201, USA

## Abstract

Our aim is to document a case of endometrioid adenocarcinoma of the ovary found in an endometriotic cyst that was suspected on pelvic ultrasound in a patient with polycystic ovary syndrome, normal Ca125, and a recent history of ovulation induction for IVF. She underwent an exploratory laparotomy with left salpingo-oophorectomy and omental biopsies followed by reexploration, complete staging, and modified radical abdominal hysterectomy and right salpingo-oophorectomy. An endometrioma described as suspicious for malignancy by an experienced ultrasound examiner should prompt immediate referral to a gynecological oncologist irrespective of Ca125 levels especially in women with a history of ovulation induction and endometriosis.

## 1. Introduction

Endometriosis is characterized by the presence of endometrial tissue and stroma outside the uterus. It is common, affecting 10%–15% of all women and an even higher percentage of infertile women (up to 47%) [1]. Unlike endometriosis, ultrasound studies are efficient for diagnosing ovarian endometriosis which is often expressed as endometriotic cyst, or endometrioma.

Malignant transformation of endometriosis is rare, but may occur in up to 1% of women, with the most common site being the ovary [2]. Indeed, stage I ovarian carcinomas have been reported to present as pelvic pain and endometriosis [3]. However, the reported increased risk of ovarian cancer in patients with polycystic ovary syndrome (PCOS) [4, 5] and endometriosis [2] may be confounded by factors such as inconsistency in diagnosis, contraceptive use and the lower parity rates observed in women with infertility [6]. In addition, there has been considerable concern about whether ovulation induction in itself is associated with an increased risk of ovarian cancer, with the observation that prolonged use of agents such as clomiphene citrate is associated with an increased risk of ovarian tumors in infertile women [7, 8]. We present a case of endometrioid adenocarcinoma of the ovary found in an endometriotic cyst first suspected on pelvic ultrasound in a patient with PCOS and a recent history of ovulation induction and IVF.

## 2. Case Presentation

A 33-year-old Asian woman returned for a frozen embryo transfer (FET) cycle three years following an uncomplicated pregnancy and birth that resulted from a previous successful attempt at ovulation induction with gonadotropins and IVF. She had been placed on an antagonist protocol and her stimulation was unremarkable. Her medical history was significant for hyperlipidemia and oligoovulation secondary to PCOS, which was diagnosed at the time of her initial presentation. At that time, she had irregular menses occurring every 1–4 months and evidence of bilateral polycystic ovaries on ultrasound. Her androgen levels were normal and she did not exhibit any evidence of clinical hyperandrogenism. Prior to returning for FET, her menses remained irregular; however she denied the use of any hormonal agents. There was no personal or familial history of endometriosis or gynecologic malignancies. Pelvic ultrasounds (PUs) performed at her initial infertility consult as well as during treatment and in the early stages of pregnancy revealed polycystic-appearing ovaries, but were otherwise normal.

**Figure 1:**
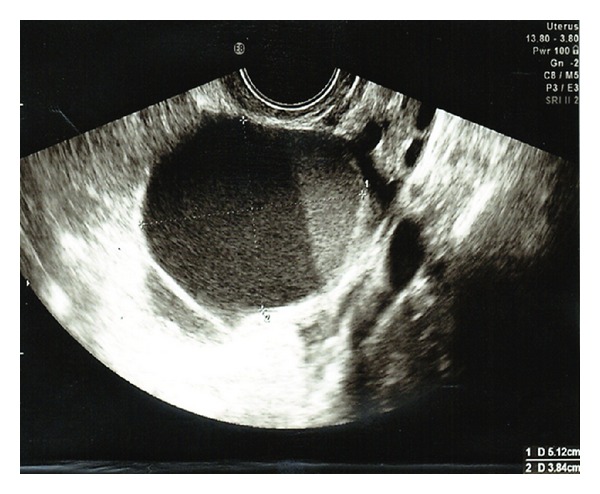
Pelvic ultrasound, showing a large complex ovarian cyst with layered low level echoes measuring 5.1 × 3.8 cm.

**Figure 2:**
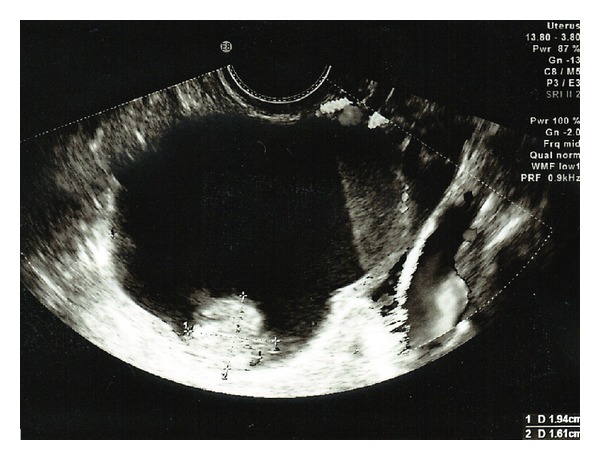
Pelvic ultrasound, showing the large complex ovarian cyst with layered low level echoes measuring 1.9 × 1.6 cm.

Prior to the commencement of a FET cycle, the patient had a baseline PU, which revealed a complex ovarian cyst with layered low level echoes measuring 5.1 × 3.8 cm (Figure 1). One area at the base of the cyst had a papillary growth (Figure 2), while color Doppler revealed venous flow within the growth suggesting malignancy. Tumor markers including CA125 (21 U/mL) were within normal range. A repeat PU and a subsequent CT scan confirmed the previous ultrasound findings.

At exploratory laparotomy by gynecological oncologist, an enlarged fluctuant and cystic left ovary adherent to the rectosigmoid was found. The pelvis was otherwise normal. After obtaining peritoneal washings, the patient underwent a left salpingo-oophorectomy and omental biopsy but the cyst inadvertently ruptured during the procedure. Gross examination showed a 5 cm cyst with a 2.5 cm papillary lesion arising from the cyst wall. Final pathology revealed a stage 1c, grade 2 intracystic endometrioid adenocarcinoma arising in an endometriotic cyst and an ovary with changes including multiple cortical follicular cysts and stromal luteinization, consistent with polycystic ovarian disease. The omental biopsy and pelvic washings were negative for malignancy.

The patient's postoperative course was uneventful. Because there was spillage of the tumor during the initial procedure, the patient desired more aggressive management. She was reexplored and underwent a modified radical hysterectomy, right salpingo-oophorectomy, pelvic and paraaortic lymphadenectomy, appendectomy, and omentectomy. Final pathology revealed no residual disease. She received 6 cycles of cytotoxic chemotherapy with carboplatin and taxol. With regard to her reproductive plans, she is considering frozen embryo transfer with a gestational carrier.

## 3. Discussion

 Risk factors for malignant transformation of endometriosis or endometriomas have not been well defined; however it is thought that estrogen exposure may facilitate the process [9]. Other factors that may be involved are concomitant PCOS and the histopathologic features of endometriosis; ectopic endometrium in endometriosis is more proliferative than eutopic endometrium and retains hormonal responsiveness, even in the postmenopausal state [10]. 

 The association between ovulation induction and ovarian cancer is biologically plausible, given that incessant ovulation during the reproductive years is a known risk factor for ovarian cancer. However, there have been no clinical trials on the effects of ovulation inducing drugs on cancer. Most of the studies published to date are retrospective, cohort, or case control studies; all have been hindered by sample size, methodology, inability to control for risk factors, and short follow-up times. When ovarian cancers occur in patients with previous use of fertility drugs, it must be questioned whether such cancers are the results of the drug exposure itself or the reasons for which the drug was prescribed. 

 Our patient is atypical in that she did not have any history or symptoms suggestive of endometriosis, neither were there gross evidences of endometriosis reported at surgery or in the pathology specimen. CA125 level was normal in our case. This is not unusual as concomitant single measurement of CA125 is unhelpful compared to diagnostic pelvic ultrasound in the preoperative evaluation of adnexal masses [11]. 

 Endometriosis-associated ovarian cancer (EAOC) usually occurs in younger women who are diagnosed more frequently at stage I, are predominantly endometrioid and clear cell histologic subtypes rather than serous, and have fewer cases of residual tumor and better survival after surgery [12]. Despite these, our patient undoubtedly underwent the best therapy for stage 1c ovarian cancer (surgery and adjuvant multiagent chemotherapy), a decision that was less difficult to make because she wanted “everything done” and expressed no further desire for fertility. 

Speculations concerning the development of ovarian cancer in women during or shortly after the use of ovulation inducing agent will continue. While such drugs may induce growth in existing highly differentiated but indolent tumors, they may also simply reflect more intensive surveillance in infertile women. Endometriomas classified as suspicious for malignancy by an experienced ultrasound examiner should prompt immediate referral to gynecological oncologist irrespective of Ca125 levels especially in women with a history of ovulation induction. Malignant transformation of endometriosis is rare; however, this case underscores the importance of careful monitoring and evaluation of adnexal masses in patients with PCOS in the setting of endometriosis, especially in infertility patients.
